# Spatial Working Memory in Male Rats: Pre-Experience and Task Dependent Roles of Dopamine D1- and D2-Like Receptors

**DOI:** 10.3389/fnbeh.2017.00196

**Published:** 2017-10-13

**Authors:** Mekite Bezu, Jovana Maliković, Martina Kristofova, Ephrem Engidawork, Harald Höger, Gert Lubec, Volker Korz

**Affiliations:** ^1^Department of Pediatrics, Medical University of Vienna, Vienna, Austria; ^2^Department of Pharmaceutical Chemistry, University of Vienna, Vienna, Austria; ^3^School of Pharmacy, College of Health Sciences, Addis Ababa University, Addis Ababa, Ethiopia; ^4^Core Unit of Biomedical Research, Division of Laboratory Animal Science and Genetics, Department of Biomedicine, Medical University of Vienna, Vienna, Austria; ^5^Paracelsus Medical University, Salzburg, Austria; ^6^Brain Research Center, Medical University of Vienna, Vienna, Austria

**Keywords:** cognition, dopamine receptor D1, dopamine receptor D2, T-Maze, working memory, water maze

## Abstract

The dopaminergic system is known to be involved in working memory processed by several brain regions like prefrontal cortex (PFC), hippocampus, striatum. In an earlier study we could show that Levodopa but not Modafinil enhanced working memory in a T-maze only during the early phase of training (day 3), whereas the later phase remained unaffected. Rats treated with a higher dose performed better than low dose treated rats. Here we could more specifically segregate the contributions of dopamine type 1- and 2- like receptors (D1R; D2R) to the training state dependent modulation of spatial working memory by intracerebroventricular (ICV) application of a D1R-like (SKF81297) and D2R-like agonist (Sumanirole) and antagonist (SCH23390, Remoxipride) at a low and high dose through 3 days of training. The D1R-like-agonist at both doses enhanced working memory at day 1 but only in the low dose treated rats enhancement persists over training compared to control rats. Rats treated with a high dose of a D1R-like-antagonist show persistent enhancement of working memory over training, whereas in low dose treated rats no statistical difference at any time point could be determined compared to controls. The D2R-like-agonist at both doses does not show an effect at any time point when compared to control animals, whereas the D2R-like antagonist at a low dose enhanced working memory at day 2. For the most effective D1R-like agonist, we repeated the experiments in a water maze working memory task, to test for task dependent differences in working memory modulations. Treated rats at both doses did not differ as compared to controls, but the temporal behavioral performance of all groups was different compared to T-maze trained rats. The results are in line with the view that spatial working memory is optimized within a limited range of dopaminergic transmission, however suggest that these ranges vary during spatial training.

## Introduction

Spatial working memory is a highly dynamic process of short-time encoding of spatial information (Dudchenko, 2004) to adjust subsequent behavior. Dopaminergic signaling in the prefrontal cortex (PFC), the hippocampus and the striatum has been identified to be involved in the modulation of working memory (Wilkerson and Levin, 1999; Miyoshi et al., 2002; Seamans and Yang, 2004; Surmeier, 2007; Yoon et al., 2008). Balanced stimulation of PFC dopamine receptors has been observed to be required for optimal working memory performance in rodents and primates (Bradley et al., 1989; Beato et al., 2008).

In a previous study (Bezu et al., 2016) we found a precise time window of working memory regulation in the presence of Levodopa, a precursor of dopamine and Modafinil an inhibitor of the dopamine transporter. Both drugs have their main impact on working memory at day 3 of a 6 day training in a delayed alternation T-maze task. Rats treated with Modafinil showed significantly better performance at low and Levodopa treated rats at high doses.

However, it remains unclear which dopamine receptors are involved in these processes and how they regulate working memory. Therefore, we used the same training protocol in the present as in the previous study, but trained the animals for 3 days only. Training was conducted in the presence of agonists and antagonists of the dopamine type 1- (D1R) and 2-like (D2R) receptors.

D1R and D2R receptors are differently distributed in the rat PFC. Generally more D1R than D2R are expressed in pyramidal and γ-aminobutyric acidergic (GABAergic) neurons with little overlap between receptor types (Santana et al., 2009). There is some evidence that D2R are diffusely distributed across cells, whereas D1R are more located in membranes (Voulalas et al., 2011). A separation between D1R and D2R receptors was also reported for the hippocampal region with highest expression in the enthorinal cortex and layer specific segregation. The cornu ammonis 1 (CA1) of the hippocampus is rich of D1 but not of D2 receptors (Köhler et al., 1991).

Dopamine has been identified to regulate working memory within a relatively narrow range of concentration and subsequent D1 and D2 receptor activation. Cortical dopaminergic transmission has been found to act in an inverted U-shaped manner, deficits in working memory can be induced by either inflated or deficient dopaminergic transmission (Zahrt et al., 1997; Cools and D’Esposito, 2011) mainly by postsynaptic effects in the PFC (Williams and Goldman-Rakic, 1995; Seamans and Yang, 2004; Vijayraghavan et al., 2007; Cools and D’Esposito, 2011). Imbalanced receptor activations can induce opposite effects on working memory compared to within-range levels (Luciana et al., 1992; Bushnell and Levin, 1993; Murphy et al., 1996; Cai and Arnsten, 1997; Wilkerson and Levin, 1999). However temporal effects, in terms of subsequent training sessions, of dopamine receptors on working memory are rarely reported. Thus, the study aimed at revealing such possible effects and whether they are receptor specific.

## Materials and Methods

### Subjects

Male Sprague-Dawley rats (12–13 weeks old), bred and maintained in the Core Unit of Biomedical Research, Division of Laboratory Animal Science and Genetics, Medical University of Vienna were used. Animals lived in the separate experimental room 1 week before and throughout the experiment. Rats were housed individually in standard Makrolon cages filled with autoclaved wood chips (temperature: 22 ± 2°C; humidity: 55 ± 5%; 12 h artificial light/12 h dark cycle: light on at 7:00 am). The study was carried out according to the guidelines of the Ethics committee, Medical University of Vienna, and were approved by the Federal Ministry of Education, Science and Culture, Austria.

### Surgery

The rats were anesthetized with Nembutal (40 mg/kg, i.p.). An intracerebroventricular (ICV) cannula (4.5 mm in length) was stereotactically implanted into the lateral ventricle of the right hemisphere (coordinates: AP—0.8; L 1.5 from Bregma). Together with an anchor screw the cannula was fixed with dental cement (Paladur, Heraeus Kulzer, Hanau, Germany). The animals were allowed to recover from surgery for at least 4 days. The correct placement of the cannulas were tested by an angiotensin II (70 ng/μl; 5 μl volume) drinking test (drinking within 3 min). From 97 rats 16 rats failed to drink, 81 rats were included in the experiment. ICV application was choosen in order to circumvent effects on peripheral dopamine receptors resulting in unwanted side effects.

### Pharmacological Treatment

The receptor agonists (D1R-like: (±)-6-CHLORO-PB hydrobromide (SKF-81297); K_i_ D1:2.2 nM; D2R-like: Sumanirole maleate; Sigma-Aldrich, S143 and SML1087, respectively); K_i:_ D2:9.0 ± 1, D3:1940 ± 142; D4:>2190, D1:>7140, nM and antagonists (D1R-like: (R+)-SCH-23390 hydrochloride (Sigma-Aldrich, D054); K_i:_ D1:0.11–0.35; D5:0.11–0.54; nM; D2R-like: Remoxipride hydrochloride (Tocris, 0916); K_i:_ D2:54–300; D3:969–1600; D4: 2800–3690 nM, were dissolved in saline and applied at a volume of 5 μl at a rate of 1 μl/min using a Hamilton syringe (CR700–20). Saline treated rats served as controls. K_i_ values were taken from Andersen and Jansen (1990); Vallone et al. (2000) for SCH-23390 and Remoxipride (min/max values), McCall et al. (2005) for Sumanirole (mean and standard error). Doses were chosen because of previous own and literature experiments that show that these doses affect learning and memory. Saline and not artificial cerebrospinal fluid was used as control substance because it was the dissolvent for the drugs.

### T-maze

The T- maze (black acrylic) consisted of two goal arms (50 cm long, 10 cm in width, with walls of 25 cm height). The starting arm (70 cm) was equipped with a starting box (20 cm in length) separated from the maze by a guillotine door. At the end of each goal arm, food pellets were provided (dustless precision pellets, 45 mg, Bio-Serv, Frenchtown, NJ, USA) in a small cup (to mask the food pellet). Food pellets were also placed outside both goal arms to mask olfactory cues. Visual cues (equipment, walls, doors) were identifiable around the maze. The maze was cleaned with 1% Incidin^®^ after the training of each individual animal in order to remove olfactory cues. Indirect illumination provided equal light intensities in each arm. Trials were monitored by a camera fixed to ceiling and videos stored at a PC.

### Procedure

#### Handling and Habituation

A total of 81 rats were used for the experiments. The rats were handled for 15 min each day for three consecutive days and the body weight was recorded daily throughout the experiment. The animals were mildly food deprived to reach a body weight of 85% of the initial weight, which was maintained during the entire experiment. Tap water was given *ad libitum*. Cage controls were also food-deprived for the same time period as trained animals and kept in their home cages in the experimental room.

Food reward pellets were provided in the home cage each day for a few days prior to training in order to familiarize to the reward. Animals were habituated to the maze until they voluntarily ate a piece of pellet placed at the end of each arm. At day 4 and 5 the animals were habituated to the maze by a 15-min free exploration of the apparatus with scattered pellets at day 4 and baited food cups only at day 5.

### Drug Administration and Training

Substances were applied at a dosage of 1 μg and 5 μg 30 min prior to each training session at days 6, 7 and 8.

A delayed none matching to place task was performed. Each training session consisted of 10 trials (a forced trial followed by nine choice trials). A trial started with the rat placed in the starting box for 15 s, after which the guillotine door was opened. In the forced trial, a randomly selected goal arm was blocked by a guillotine door, and a reward was placed in the opposite arm, hence the rats were forced to visit a baited arm.

In choice trials, both arms were accessible, but reward was available only in the arm not entered in the previous trial. In the choice trials 1 through 9, rats had to avoid the arm visited in the previous trial and received the reward in the opposite arm. The intertrial interval was 5 min. After choosing an arm, the rat was allowed to consume the pellet within 10 s. Arm entries were recorded when the whole animal, including the tail tip, was in the arm. If rats selected the un-baited arm, a self-correction procedure was introduced by keeping the baited arm baited until it was visited, giving the rats a chance to shift their choice. Entry into the arm visited in the previous trial was registered as an error of working memory. In addition, a working memory index was calculated (correct choices/total trials).

### Water Maze

The maze consisted of a black circular plastic pool (150 cm in diameter, 60 cm wall height) equipped with a black quadratic escape platform (10 cm × 10 cm) at a height of 38 cm. The pool was filled with water (25 ± 2°C) up to 39.5 cm. Platform positions were located halfway between the wall and the middle of the pool in four quadrants. Five different positions were used each day for the sample trials (90 s to discover the platform) followed 5 min later by a test trial (90 s) for recall of the platform position. In case the animals did not find the platform during the sample trial, they were guided by hand and allowed to remain for 15 s onto it. Starting positions for sample and test trials varied pseudorandomly. Before the 3 day training phase rats were handled 2 days for 15 min each, and thereafter habituated to the pool at the following day by a 90 s swim without platform. The time to reach the platform (escape latency), the distance traveled and the mean velocity was recorded by a tracking system (TIBE, V 1.0, Imagination, Vienna, Austria) and stored on a computer. Similar as for the T-maze animals were infused ICV with the DIR agonist at a dose of 1 μg, 5 μg and saline controls 30 min prior to training. For the analysis the mean of escape latencies and velocities of the five test trials for each rat/day was calculated. These values were then statistically analyzed between groups.

All behavioral training/testing was performed during the light phase of the light–dark cycle.

### Statistics

A repeated measure ANOVA with Tukey *post hoc* tests for the differences over the entire training and Bonferroni *post hoc* tests for differences at specific days was conducted. Within group performance over training days were tested by *T*-test for connected samples. Border of significance was set at *p* ≤ 0.05.

## Results

### T-maze

**Figure 1:**
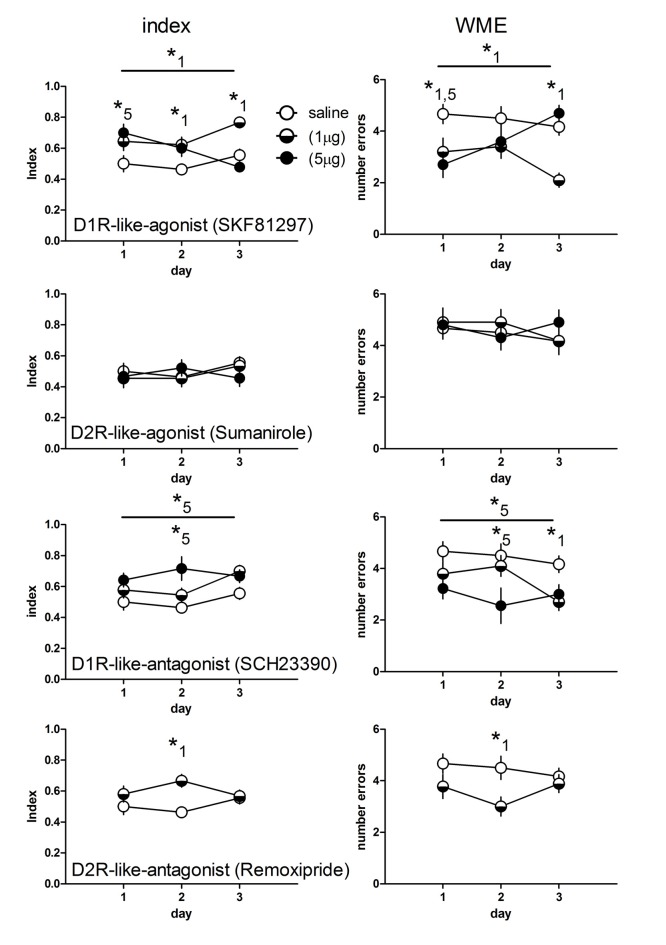
Working memory indices (left panel) and numbers of working memory errors (WME, right panel) of rats treated with an D1- (*n* = 10 each) and D2-like agonist (1 μg: *n* = 11; 5 μg: *n* = 10) for each dose) and antagonist (D1: 1 μg: *n* = 10; 5 μg: *n* = 9; D2: *n* = 9) or saline (*n* = 12). Significant differences between groups over the entire training are indicated by an asterisk above horizontal bars. Significant differences between groups for specific days are indicated by asterisks above daily data points. Numbers give the drug dose (1: 1 μg; 5: 5 μg). Given are the mean values and SEM.

All groups were included in the statistical analysis but the results are presented separately in Figure 1 for clarity. There was a significant day × treatment interaction (*F*_(14,146)_ = 2.27, *p* = 0.008) and a significant treatment effect (*F*_(7,73)_ = 5.79, *p* < 0.001) for the working memory index. Similarly the analysis of the working memory errors (WME) revealed a significant day × treatment interaction (*F*_(14,146)_ = 2.17, *p* = 0.011) and a significant treatment effect (*F*_(7,73)_ = 5.75, *p* < 0.001).

### Effects of the D1R-Like Agonist

D1R-like agonist treated rats at a dose of 1 μg showed higher working memory indices throughout the training as compared to saline treated (*p* = 0.007), whereas a 5 μg dosage yielded no difference to control animals (*p* = 0.56). Day specific analysis revealed a significant higher index in rats treated with 5 μg (*p* < 0.01) at day 1 but not day 2 and 3. Rats treated with 1 μg show higher indices than controls at day 2 (*p* < 0.05) and day 3 (*p* < 0.01).

Considering the WME D1R-like agonist (1 μg) treated rats performed less errors than controls over training (*p* = 0.008) and specifically at day 1 (*p* < 0.05) and day 3 (*p* < 0.01), whereas rats treated with 5 μg did not show less errors than controls (*p* = 0.56) over training but significantly reduced errors at day 1 (*p* < 0.01).

### Effects of the D2R-Like Agonist

Agonists for D2-like receptors did not induce differences compared to controls over training (index and errors, 1 μg and 5 μg: *p* = 0.99 each) and no differences at any day (*p* > 0.05, each).

### Effects of the D1R-Like Antagonist

Indices and WME of D1R-like antagonist treated rats at a dose of 1 μg did not differ compared to controls over training (*p* = 0.35, each) and no index differences at any day (*p* > 0.05, each) but significantly reduced errors at day 3 (*p* < 0.05), whereas rats treated with 5 μg showed significantly enhanced indices and reduced errors over training (*p* = 0.013, each) and specifically at day 2 (*p* < 0.001).

### Effects of the D2R-Like Antagonist

Because we did not find differences for the D2R-like agonist, we treated rats with the antagonist at only one dosage (1 μg). We found no overall difference for the index and the errors (*p* = 0.42, each) compared to controls. However the *post hoc* tests for days revealed significant enhanced indices (*p* < 0.01) and reduced errors (*p* < 0.05) at day 2.

### Water Maze

Water maze experiments were done only for D1R-like agonist treated rats, since this was the most effective drug in the T-maze experiments and therefore served for testing task dependent effects.

**Figure 2:**
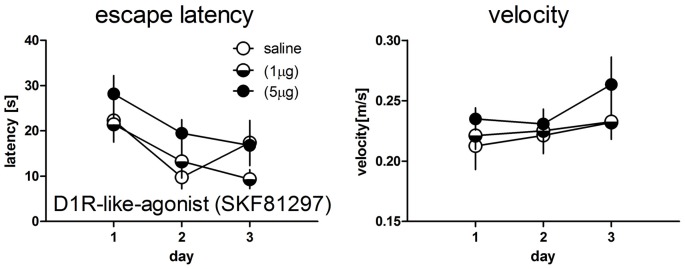
Escape latencies (left panel) and mean velocity (right panel) during test trials in the water maze working memory task in rats treated with saline (*n* = 9) or the D1R-like-agonist at a dose of 1 μg (*n* = 10) or 5 μg (*n* = 8). No significant differences between groups could be detected. Given are the mean values and SEM.

There was no significant day × treatment interaction (*F*_(4,48)_ = 1.43, *p* = 0.239) and no significant treatment effect (*F*_(2,27)_ = 1.46, *p* = 0.253) for the escape latencies as well as for the swim velocity (*F*_(4,48)_ = 0.42, *p* = 0.796) and (*F*_(2,24)_ = 0.73, *p* = 0.493), respectively (Figure 2).

### Within-Group Performance Over Training Days

Within group performance in D1R-like agonist treated rats between days differed task dependently. Performance in the T-maze control group (day 1–day 2: *T* = 0.63, *p* = 0.54; day 1–day 3: *T* = −1.15, *p* = 0.27; day 2–day 3: *T* = − 1.89, *p* = 0.085, *df* = 11) as well as for the low dose treated rats (day 1–day 2: *T* = 0.29, *p* = 0.78; day 1–day 3: *T* = −1.67, *p* = 0.13; day 2–day 3: *T* = − 2.25, *p* = 0.051, *df* = 9) did not differ between days. High dose treated rats performed significantly worse at day 3 compared to day 1 (*T* = 3.46, *p* = 0.007) but not between the other days (day 1–day 2: *T* = 1.09, *p* = 0.30; day 2–day 3: *T* = 1.77, *p* = 0.11, *df* = 9).

Performance in the water maze control rats (day 1–day 2: *T* = 3.06, *p* = 0.016; day 1–day 3: *T* = −1.36, *p* = 0.21; day 2–day 3: *T* = −2.37, *p* = 0.045, *df* = 8) and low dose treated rats (day 1–day 2: *T* = 3.39, *p* = 0.008; day 1–day 3: *T* = −4.52, *p* = 0.001; day 2–day 3: *T* = 1.17, *p* = 0.27, *df* = 9) differed as compared to T-maze tested rats. High dose treated rats did not show significant differences in daily performance (day 1–day 2: *T* = 1.65, *p* = 0.14; day 1–day 3: *T* = 2.10, *p* = 0.073; day 2–day 3: *T* = 0.57, *p* = 0.58, *df* = 7).

### Comparison of D1R-Like Agonist Effects between the Two Tasks

**Figure 3:**
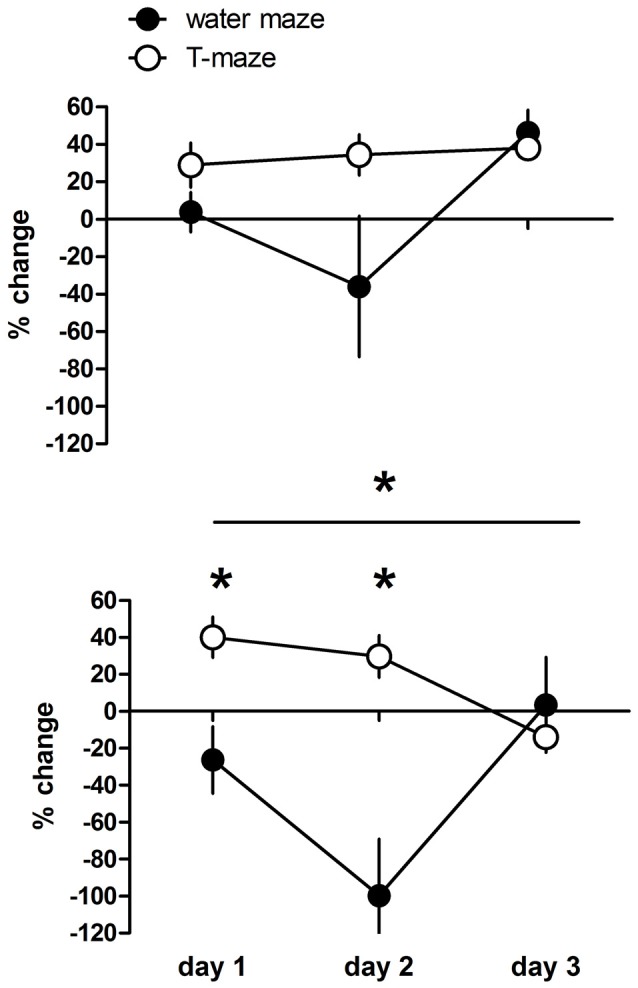
Changes in working memory indices (T-maze) and escape latencies (water maze) expressed as variation in percentage from the mean value (100%) of the respective control group of individual D1R-like agonist treated rats at a dose of 1 μg or 5 μg. Positive values indicate improvement and negative deterioration of working memory. Significant differences between groups over the entire training are indicated by an asterisk above horizontal bars. Significant differences between groups for specific days are indicated by asterisks above daily data points. Given are the mean values and SEM.

Direct comparison between drug effects between the two tasks is not possible due to the different units of behavioral recording. Therefore, we compared the deviation of the performance of individual drug treated rats expressed as percentage from the mean performance of the respective control group between rats trained in the T-maze or water maze (Figure 3). Given are the changes in working memory indices (T-maze) and escape latencies (water maze). Positive values indicate improvement and negative deterioration of working memory. The overall analysis revealed a significant day effect (*F*_(2,68)_ = 5.59, *p* = 0.006), a day × task interaction (*F*_(6,68)_ = 4.79, *p* < 0.001) and a significant task effect (*F*_(3,34)_ = 6.60, *p* = 0.001). *Post hoc* tests revealed no significant difference between T-maze and water maze trained rats treated with the low dose (*p* = 0.307), whereas high dose treated rats performed less in the water maze compared to the T-maze (*p* = 0.009), specifically at day 1 (*p* < 0.05) and day 2 (*p* < 0.001) but not at day 3 (*p* > 0.05).

## Discussion

We found different dopamine receptor activation dependent modulations of spatial working memory in subsequent training sessions in the T-maze differing in a receptor specific manner. D1R like mechanisms could be determined as main modulator with increasing memory performance at a low dose of the agonist and at a high dose of the antagonist, whereas D2R like activity is less involved with a day specific effect only for the antagonist. The results are in line with the view that spatial working memory is optimized within a limited range of dopaminergic transmission (Aultman and Moghaddam, 2001; Williams and Castner, 2006; Avery and Krichmar, 2015).

Increased spontaneous alternation in a Y-maze was induced in the presence of the dopamine antagonist SCH-23390 (Rusu et al., 2014). Similar to our study working memory was mainly modulated by D1R but not D2R blockade. In contrast to the present study working memory was impaired at all drug concentrations and all test delays used, however session (day) specific effects were not determined (Bushnell and Levin, 1993; Wilkerson and Levin, 1999). Dose related impairments of spatial working memory by intra-PFC injections of D1R receptor agonists have been found (Williams and Goldman-Rakic, 1995) that were attenuated by pretreatment with an D1R antagonist (Zahrt et al., 1997), however no session specific effect was analyzed.

Our results suggest that the optimal range of receptor activation may vary according to the preexperience of the animals, e.g., the training status in the T-maze. D1R receptor agonist infusions into the PFC resulted in a memory strength dependent effect. Stronger memories (short delay) were disrupted, whereas weaker memories (long delay) were improved at all used drug concentrations (Floresco and Phillips, 2001). The functional interplay between different working memory related brain regions and region specific optimal ranges may contribute to our result of session specific differences of responses to the drug treatment. Especially the communication between the PFC and basal ganglia determine working memory (Gruber et al., 2006; van Schouwenburg et al., 2010). Differences in the optima of dopamine concentrations and receptor specific transmissions within these areas during training may therefore explain our results (Seamans et al., 1998; Chudasama and Robbins, 2004). Especially day 2 performance is sensitive for the blockade of both D1- and D2-like receptors, suggesting that at this training stage a dopaminergic overstimulation impairs working memory, this is further supported by the smaller agonist effect on this day. Therefore, dopaminergic inhibition at this stage may protect against internal noise (Williams and Castner, 2006; Avery and Krichmar, 2015) and enhance working memory.

However, the D1R like dependent modulation of working memory is task dependent, since we found no significant differences between treated and control rats in the water maze task. This may be related to different reasons, first the water maze working memory task is D1R independently regulated. This seems to be unlikely because prefrontal D1 dependence of working memory in the water maze has been shown in D1R mutant mice (Xing et al., 2012) and at least dopamine dependence in rats (Wisman et al., 2008; Murphy et al., 2015). Another reason may be the endogenous changes of the dopaminergic system due to the difference in day to day performance making the system less sensitive for exogenous treatment. Day to day performance differed between T-maze and water maze trained rats in controls but also dose dependently, with a rapid improvement from day 1 to day 2 in control and low dose but not high dose treated rats in the latter, whereas T-maze treated rats did not show significant differences between days. Thus the working memory underlying mechanisms (but not that of specific memories) and circuitries may be rapidly strengthened in water maze trained rats making additional activations by the agonist less effective and therefore cause no differences between control and treated animals. Strengthening remains in low dose treated rats at day 3 three whereas in controls performance at day 3 returns to day 1 levels. Thus, similar to the T-maze day 2 seems to be a crucial sensitive time point for working memory performance. Further, working memory in the water maze may be related by different brain structures and cell types as in the T-maze. The first but not the second depends on food-reward and the second is more stressful for the animals (Korz and Frey, 2004). Thus, it is likely that the second in contrast to the first involves the amygdala (Zancada-Menendez et al., 2017). Related conclusions that can be drawn by the present study are limited and underlying mechanisms have to be revealed in further studies. Task dependent differences could be observed only for the high dose D1R-like agonist treated rats exhibiting deterioration of working memory at day 1 and 2 in the water maze compared to the T-maze with the most pronounced effect at day 2, which again point to a time dependent sensitivity and restructuring of the dopaminergic system.

The treatment itself may also change the molecular basis of processing of working memory in a brain region and dose dependent manner. Increased receptor internalization by the application of specific receptor agonists has been reported for D2 (Goggi et al., 2007) and D1 receptors (Dumartin et al., 1998). Therefore, the dopamine receptor subtype composition may change due to these processes and can contribute to the observed changes in working memory performance. The training related changes at the neuronal, molecular and receptor processes can interfere with the pharmacological treatment and therefore contribute to the observed temporal changes in working memory performance (Klingberg, 2010; Buschkuehl et al., 2012; Söderqvist et al., 2012). At present we cannot localize specific mechanisms due to the limited experimental approach. However, our results are in line with others that working memory is modulated by optimal dopaminergic transmission, and in addition suggest that these optimal ranges can change during the training due to the changed pre-experience probably at the cognitive, molecular and physiological level not only between tasks but also within tasks. Future evaluation of the underlying mechanisms, as well as the involved brain regions and cell types, may contribute to the revelation of dopaminergic modulation of working memory and task dependent optimization of cognitive enhancing pharmacological treatment (Trossbach et al., 2014).

## Author Contributions

MB, JM and MK did the experimental work; EE and HH revised the manuscript, GL and VK designed the experiments and wrote the manuscript.

## Conflict of Interest Statement

The authors declare that the research was conducted in the absence of any commercial or financial relationships that could be construed as a potential conflict of interest.
